# Creep Rupture of the Simulated HAZ of T92 Steel Compared to that of a T91 Steel

**DOI:** 10.3390/ma10020139

**Published:** 2017-02-08

**Authors:** Yu-Quan Peng, Tai-Cheng Chen, Tien-Jung Chung, Sheng-Long Jeng, Rong-Tan Huang, Leu-Wen Tsay

**Affiliations:** 1 Institute of Materials Engineering, National Taiwan Ocean University, Keelung 20224, Taiwan; satan-sammael@yahoo.com.tw (Y.-Q.P.); k25856545@hotmail.com (T.-J.C.); rthuang@mail.ntou.edu.tw (R.-T.H.); 2 Division of Nuclear Fuels and Materials, Institute of Nuclear Energy Research, Lungtan, Taoyuan 32546, Taiwan; tcchen@iner.gov.tw (T.-C.C.); sljeng@iner.gov.tw (S.-L.J.)

**Keywords:** T92 steel, simulated HAZ, creep rupture, post-weld heat treatment

## Abstract

The increased thermal efficiency of fossil power plants calls for the development of advanced creep-resistant alloy steels like T92. In this study, microstructures found in the heat-affected zone (HAZ) of a T92 steel weld were simulated to evaluate their creep-rupture-life at elevated temperatures. An infrared heating system was used to heat the samples to 860 °C (around A_C1_), 900 °C (slightly below A_C3_), and 940 °C (moderately above A_C3_) for one minute, before cooling to room temperature. The simulated specimens were then subjected to a conventional post-weld heat treatment (PWHT) at 750 °C for two hours, where both the 900 °C and 940 °C simulated specimens had fine grain sizes. In the as-treated condition, the 900 °C simulated specimen consisted of fine lath martensite, ferrite subgrains, and undissolved carbides, while residual carbides and fresh martensite were found in the 940 °C simulated specimen. The results of short-term creep tests indicated that the creep resistance of the 900 °C and 940 °C simulated specimens was poorer than that of the 860 °C simulated specimens and the base metal. Moreover, simulated T92 steel samples had higher creep strength than the T91 counterpart specimens.

## 1. Introduction

The increased application of ultra-supercritical (USC) power plants to reduce CO_2_ emissions and save fossil fuels has driven the development of advanced creep-resistant alloy steels. Tempered 9-12 Cr steels are favored for high temperature applications such as boiler and turbine components in fossil fuel power plants, due to their excellent combination of mechanical and oxidation-resistant properties at high temperature. T/P92 steel, containing approximately 9Cr-0.5Mo-1.8W with minor additions of V, Nb, B and N, is one of the major alloys used for components in USC power plants [1]. The addition of B into 9Cr ferritic steel delays the softening or coarsening of the M_23_C_6_ carbides [2,3] and suppresses grain refinement in the heat-affected zones (HAZ) of the weld [4]. With the addition of tungsten into the 9Cr-Mo steel, the stabilized M_2_X carbonitrides (M = Cr, Fe; X = C, N), uniform distribution of fine M_23_C_6_ carbides, and the retardation of dislocation recovery after tempering at 750 °C resulted in an enhanced high temperature tensile strength [5]. Increasing the tungsten concentration in tempered 9Cr-W steels has also been found to decrease the coarsening rate of martensite lath [6]. The substitution of W for Mo has proven to be very effective in enhancing the creep rupture strength of the HAZ of the 9Cr steel welds [7].

Prior studies have shown that the normalizing temperature greatly influences the mechanical properties of 9Cr-Mo steel [8,9,10,11]. Increasing the normalizing temperature from 850 to 1050 °C causes a decrease in the minimum creep rate and an increase in the creep life of modified 9Cr-1Mo steel [8]. At the same tempering temperature, increasing the normalizing temperature will decrease the hardness [9] and impact toughness of Gr. 91 steel at room temperature [10]. To achieve optimal mechanical properties, T92 steel should be normalized at 1060 °C and then tempered at 790 °C [11]. Compared with ASTM A335 P91 steel, P92 steel exhibits an increase of approximately 25% creep resistance at 600 °C for 10^5^ h and has corrosion and oxidation resistance characteristics of 9% Cr steels [12,13].

Welding is unavoidable in the construction of boilers for fossil fuel power plants. With proper preheating, interpass temperature control, and post-heating, cracks in P92 steel welds can be avoided after welding [14,15]. A full martensitic transformation in T/P92 weld joints by cooling to below 100 °C is required before post-weld heat treatment (PWHT) [16]. A PWHT in the temperature range of 740–780 °C is suggested to restore the ductility and impact toughness of the P92 steel weld [14,15]. According to the thermal history experienced at each microscopic site away from the fusion boundary, particular microstructures are generated in a 9Cr weld at a specific site [17]. The HAZs of a weld are generally classified as: coarse-grained HAZ (CGHAZ), reaching a temperature well above A_C3_; fine-grained HAZ (FGHAZ) heated to a peak temperature slightly above A_C3_; the intercritical HAZ (ICHAZ) heated to a peak temperature (*T*_p_) of A_C1_ < *T*_p_ < A_C3_; and the highly over-tempered zone (OTHAZ) heated to a peak temperature around A_C1_. The highest impact toughness in terms of the highest upper shelf energy and lowest ductile-to-brittle transition temperature is obtained in the ICHAZ of a 9Cr-1Mo steel weld [18], while the CGHAZ is reported to have the lowest toughness in such welds [19].

The creep properties of weld joints have been found to be inferior to those of the base and weld metals. The creep damage and crack initiation in the HAZ of P91 [3,20,21,22,23,24], P92 [4,25,26,27,28,29], and P122 steel welds [2,26,30,31] is referred to as Type IV cracking. The weakened HAZ is considered to be a serious problem for the long-term service of tempered 9-12 Cr steel welds at elevated temperatures [32,33,34]. The minimum hardness in a Gr. 91 steel weld shifts from the tempered HAZ around A_C1_ to FGHAZ during creep, and is associated with creep void formation [21]. Reducing the width of the HAZ by electron beam welding would effectively improve the creep rupture strength of 9-12 Cr steel welds [26]. However, the FGHAZ of the P92 steel weld exhibits a faster creep crack growth rate at 650 °C as compared to other micro-zones specimens [35]. With crack growth across the P92 weld metal to the base metal (BM), the fatigue crack growth rate at 600 °C increases as the crack grows into the HAZ, and then decreases as the crack propagates further into the BM [36].

The continuous microstructural changes that occur within a narrow width in the HAZ of a weld complicate the evaluation of creep properties of particular microstructures. Therefore, the microstructures and characteristics of the HAZ need to be investigated using thermal simulated specimens, which are produced by heating the samples to a specific temperature and then subjecting them to creep tests. The aim of this study was to investigate the effects of simulated microstructures on the short-term creep rupture of T92 steel welds at elevated temperatures. After a PWHT at 750 °C for two hours, all simulated samples were subjected to constant load creep tests at different temperatures.

## 2. Material and Experimental Procedures

The chemical composition (wt %) of the T92 tube used in this study is as follows: 0.12 C; 0.43 Mn; 0.23 Si; 0.014 P; 0.002 S; 8.89 Cr; 0.47 Mo; 1.76 W; 0.24 Ni; 0.20 V; 0.05 Nb; 0.003 B; 0.038 N; 0.003 Ti; with a balance of Fe. A DIL 805A/D dilatometer (TA Instruments, Hüllhorst, Germany) was used to determine the transformation temperature of this alloy. The A_C1_ and A_C3_ temperatures were measured at specific heating rates with the thermal cycle of heating the samples from room temperature to 1050 °C. After the peak temperature was reached, Ar-assisted cooling was applied to achieve different cooling rates to determine the *M*_S_ and *M*_f_ temperatures of the T92 steel. The microstructures in different regions of the HAZ were simulated by heating the as-received steel plate to the pre-determined temperature with an infrared heating system that allowed rapid heating and controlled cooling [37]. The heating rates of the simulated specimens were as high as 60 °C/s. A specific thermal history was imposed on the sample to simulate the microstructures at a particular site in the HAZ of the weld. Various microstructures in the HAZ were simulated by heating the specimens to 860, 900, and 940 °C for one minute, before cooling to room temperature. The simulated microstructures corresponded to the following:

- short-time over-tempering of the alloy (860 °C, STOT)
- heated until slightly below the A_C3_ temperature (900 °C, ICHAZ)
- fine-grained microstructures heated until moderately above the A_C3_ temperature (940 °C, FGHAZ)

Furthermore, the simulated specimens were subjected to conventional post-weld heat treatment (PWHT) at 750 °C/2 h.

**Figure 1:**
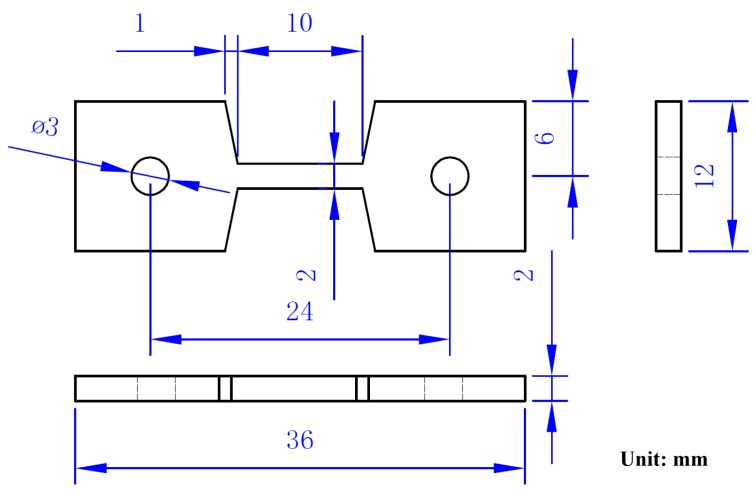
Schematic diagram showing the dimensions of the specimen for the short-term creep test.

A MVK-G1500 Vickers hardness tester (Mitutoyo, Kawasaki, Japan) was applied with a load of 300 g for 15 s to measure specimen hardness in the as-treated or PWHT conditions. The reported hardness values were averaged from eight measurements. To understand the influence of the microstructures on the failure of the simulated specimens at elevated temperature, simulated specimens after PWHT were subjected to creep-rupture tests loaded by dead weight under different conditions. The specimen dimensions for the creep-rupture test are shown in Figure 1, which were cut from a 2-inch tube with a wall thickness of 3/8 inch by an electro-discharged wire cutter. The microstructures of various specimens were inspected by BX51 optical microscope (OM, Olympus, Tokyo, Japan) and JSM-7100F field emission scanning electron microscope (SEM, JEOL, Tokyo, Japan). The specimens were also examined by an SEM equipped with NordlysMax^2^ electron backscatter diffraction detector (EBSD, Oxford Instruments, Abingdon, UK) to reveal the differences in grain sizes between the specimens. The detailed microstructures of simulated specimens were inspected by JEM-2000EX transmission electron microscope (TEM, JEOL, Tokyo, Japan).

## 3. Results

### 3.1. Microhardness Measurements

Table 1 lists the transformation temperature of T92 steel determined by a dilatometer at specific heating/cooling rates. The results indicate that the A_C1_ and A_C3_ temperatures of the T92 steel increased with an increased heating rate. At a heating rate of 0.5 °C/s, the A_C1_ and A_C3_ temperatures were 869 °C and 921 °C, respectively, and these were regarded as near-equilibrium transformation temperatures. The A_C1_ and A_C3_ temperatures at the heating rate of 60 °C/s rose to 914 °C and 962 °C, respectively. In contrast, the *M*_S_ and *M*_f_ temperatures of T92 steel shifted slightly to a lower temperature range with an increased cooling rate. At the low cooling rate of 5 °C/s, the *M*_S_ and *M*_f_ temperatures of T92 steel were as high as 392 °C and 229 °C, respectively. An increase in cooling rate to 45 °C/s reduced the corresponding *M*_S_ and *M*_f_ temperatures down to 374 °C and 195 °C. Nevertheless, the austenite would transform into martensite completely after cooling to room temperature under normal welding or heat treatment conditions.

Table 2 lists the Vickers hardness values of the specimens in the as-simulated and PWHT conditions. The BM specimens had an original hardness of Hv 245 and showed a small change in hardness after PWHT. The specimens heated to 860 °C exhibited a minor decrease in hardness to Hv 231. It was deduced that short-time over-tempering (STOT) at 860 °C would cause minor changes in the microstructure of T92 steel. After PWHT, the hardness of the STOT specimens decreased to Hv 228. Moreover, the specimen heated to 940 °C (FGHAZ) had a hardness of Hv 400, which was significantly higher than that of the other specimens. After PWHT, tempering effectively reduced the hardness of the FGHAZ specimen to Hv 242. In the case of the specimen heated to 900 °C (ICHAZ), the hardness of the as-treated sample was Hv 352. This indicated incomplete hardening, and the solution treatment at 900 °C (ICHAZ) should be below the A_C3_ temperature of T92 steel. The hardness of the tempered ICHAZ specimen was lowered to Hv 223. With the PWHT, the discrepancy in hardness between the FGHAZ and BM specimens was minor, but the ICHAZ specimen was a little softer than the other samples after tempering.

### 3.2. Microstructural Observations

**Figure 2:**
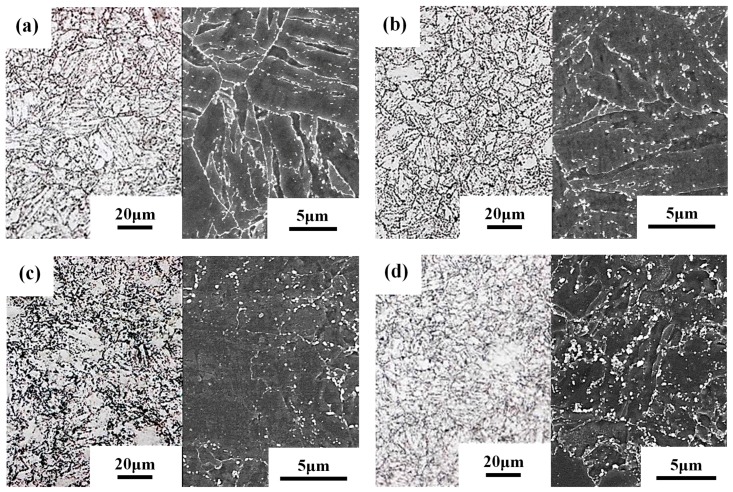
Optical (left) and SEM (right) micrographs of the: (**a**) BM; (**b**) STOT; (**c**) ICHAZ; and (**d**) FGHAZ specimens after PWHT.

**Figure 3:**
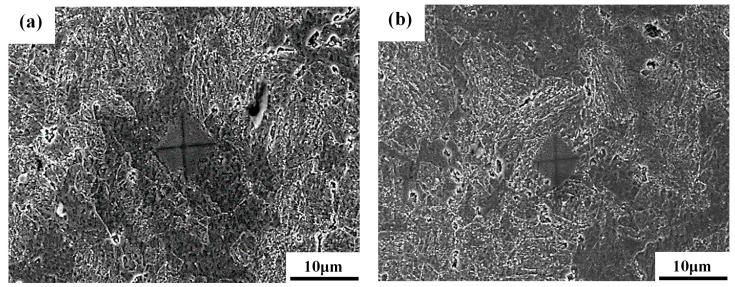
The hardness indentation on the ICHAZ specimen showing the differences in hardness in: (**a**) fine ferrite and (**b**) martensite.

Figure 2 shows the microstructures of various specimens after PWHT with an optical micrograph (on the left) and an SEM metallograph (on the right) of a representative specimen. On the whole, the BM and STOT specimens (Figure 2a,b) showed similar microstructures; i.e., the prior austenite boundaries were decorated with precipitates, and aligned precipitates were present in the tempered lath martensite. The difference between them was the slight degradation of the lath structure and agglomeration of precipitates in the STOT specimen. Furthermore, the optical micrographs of the ICHAZ (Figure 2c) and the FGHAZ specimens (Figure 2d) revealed refined microstructures. The morphologies of the lath martensite in both specimens were difficult to resolve (Figure 2c,d). The SEM micrograph showed a fine grain size, degraded lath morphology, and non-uniform distribution of the precipitates in the ICHAZ specimen (Figure 2c on the right). The micro-hardness indentation test under a load of 10 g for 10 s revealed discrepancies in the hardness in different etched zones in the ICHAZ specimen in the as-treated condition, as shown in Figure 3. It was deduced that the ICHAZ specimen was probably composed of fine ferrite subgrains (Figure 3a) and fresh martensite (Figure 3b), leading to non-uniform hardness and incomplete hardening. The occurrence of carbide dissolution and dislocation annihilation during the thermal cycle assisted the formation of carbide-free ferrite in the ICHAZ specimen. Moreover, precipitate-coarsening and agglomeration were observed in the tempered FGHAZ specimen (Figure 2d on the right).

**Figure 4:**
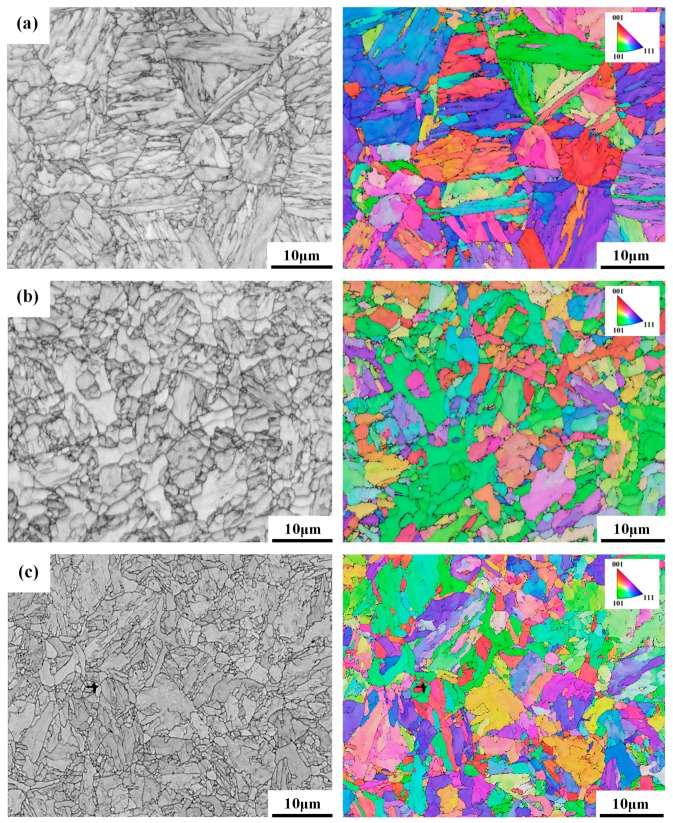
SEM micrographs (left) and electron backscatter diffraction (EBSD) maps (right) of the: (**a**) STOT; (**b**) ICHAZ; and (**c**) FGHAZ specimens after PWHT.

Figure 4 displays the microstructures of the specimens with an EBSD map showing the individual grain orientations relative to their surroundings. A great discrepancy in color between adjacent grains indicated a great difference in orientation between them. The results showed that within one grain, the martensite packages oriented in the same direction were of the same color, whereas specific colored zones related to the lath martensite in different orientations. Overall, the BM and STOT (Figure 4a) specimens after the PWHT revealed similar characteristics; i.e., equiaxial grains with lath martensite packages. In contrast, the ICHAZ specimen (Figure 4b) consisted of newly nucleated fine grains with some subgrains inside a coarse grain. The EBSD map also showed the non-uniform distribution of grain sizes and nucleated fine grains in the FGHAZ specimen (Figure 4c), most likely due to the short-time-heating above the A_C3_ temperature.

### 3.3. Transmission Electron Microscopy

**Figure 5:**
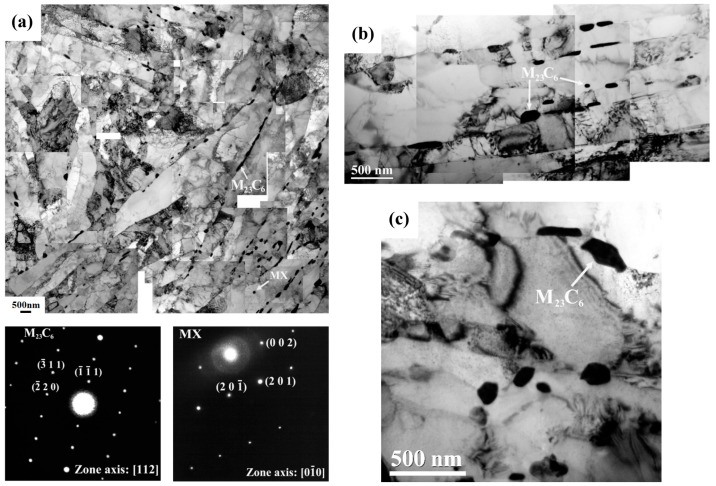
TEM micrographs of (**a**) BM with diffraction patterns of different carbides; (**b**) BM; and (**c**) STOT specimens in the PWHT condition.

Figure 5 presents TEM micrographs of the BM and STOT specimen after PWHT. Both specimens displayed quite similar microstructures; i.e., lath boundaries and prior austenite grain boundaries were decorated with precipitates (Figure 5a). The results indicated that the precipitates along the lath and austenite grain boundaries were M_23_C_6_ carbides, and was confirmed by the diffraction pattern. Some fine precipitates inter-dispersed within the martensite lath were MX carbides or carbonitrides. The Nb(CN), MX, and M_23_C_6_ precipitates were obtained in the T92 steel after normalizing and tempering at 750 °C [5,11]. It was noticed that there was a great difference in M_23_C_6_ carbide size observed at the lath boundaries, as shown in Figure 5b. However, some differences in the microstructures between the BM and STOT specimens were noted. The short-time over-heating caused the excess recovery of dislocations, the formation of new subgrains, carbide-spheroidizing, and carbide-coarsening in the STOT specimen (Figure 5c). The local breakdown of the martensite package in the STOT specimen made the lath structure less prominent than that in the BM. It was noticed that excessively coarse carbides, with a size of about 350 μm were harmful to its creep properties when compared to the BM specimen.

**Figure 6:**
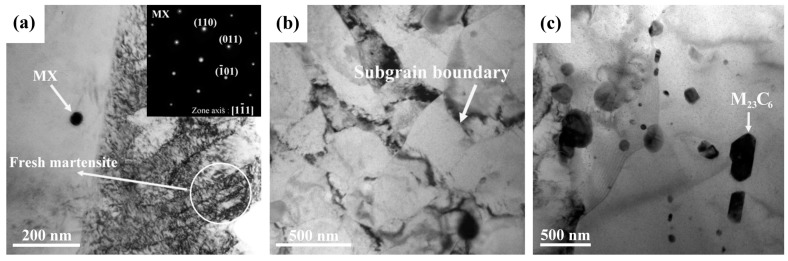
TEM micrographs of the ICHAZ specimen in the: (**a**) as-treated and (**b**,**c**) PWHT conditions.

TEM micrographs of the specimens heated to 900 °C in the as-treated and PWHT conditions are shown in Figure 6. In the as-treated condition, fresh lath martensite with a high dislocation density was observed to coexist with un-dissolved MX carbides (Figure 6a). MX precipitates were reported to undergo a negligible change in simulated P91 steel heated to a peak temperature of 1050 °C [38] and in an A_C3_ simulated HAZ of P92 steel [26]. The increase in hardness (Hv 352) of the ICHAZ specimen resulted from the formation of fresh martensite containing very fine laths. After PWHT, fine subgrains were found to be deficient of M_23_C_6_ carbides at the boundaries and lath morphology in the ICHAZ specimen (Figure 6b). As shown in Figure 6c, the excessively coarse carbides in the tempered ICHAZ specimen could be related to the rapid growth of prior un-dissolved M_23_C_6_ carbides during the PWHT. The trace of aligned carbides in a straight line implied the location of a prior boundary.

**Figure 7:**
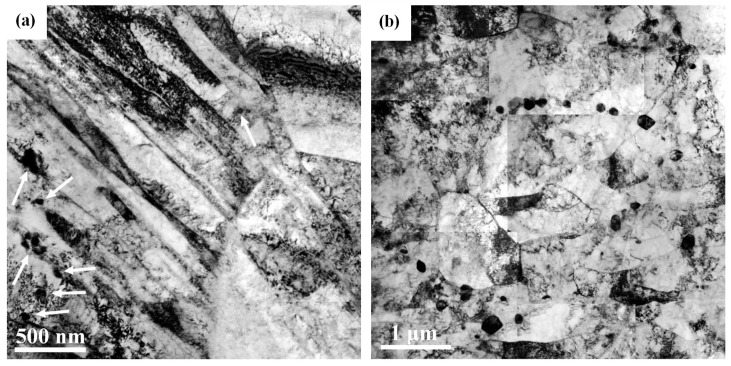
TEM micrographs of the FGHAZ specimen in the: (**a**) as-treated and (**b**) PWHT conditions.

In the FGHAZ specimen, lath martensite with a high dislocation density was found after cooling to room temperature (Figure 7a). Owing to the short-time solution treatment by infrared heating, few residual M_23_C_6_ carbides were presented at the lath boundaries (indicated by the arrows) of the un-tempered specimen (Figure 7a). After PWHT, the spheroidized M_23_C_6_ carbides of different sizes were located mainly along the prior austenite grain boundaries (Figure 7b). Furthermore, nucleated subgrains without an internal lath structure were seen in the samples; this microstructure being obviously different to that in the BM (Figure 5a).

### 3.4. Short-Term Creep Tests

**Figure 8:**
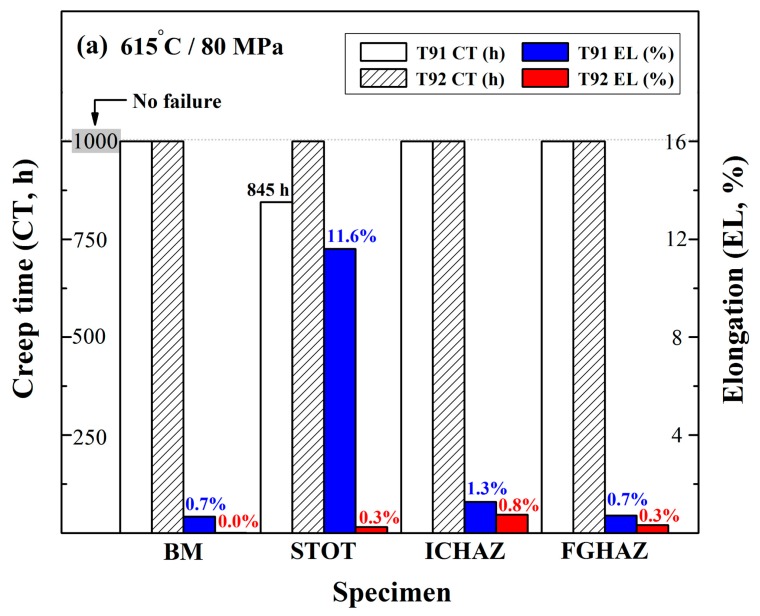

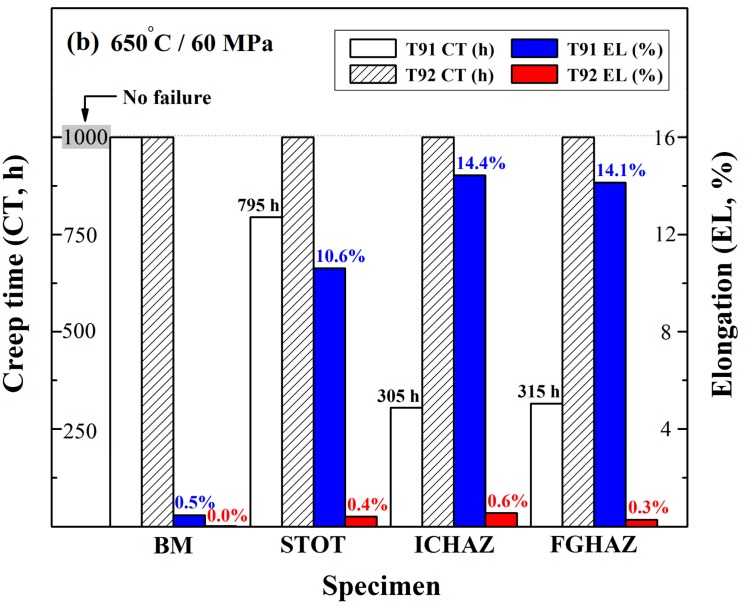
Creep tests of T91 and T92 specimens after PWHT at: (**a**) 615 °C/80 MPa; and (**b**) 650 °C/60 MPa.

Figure 8 shows the results of creep tests of T92 specimens in comparison with their T91 counterpart specimens under the testing conditions of 615 °C/80 MPa or 650 °C/60 MPa [37]. The short-term creep tests were terminated after 1000 h duration. Additionally, the elongation of the crept specimen was measured as an index for evaluating the creep resistance of the specimen. It should be noted that in Figure 8, the white and slash column indicate the creep time (CT) of T91 and T92, respectively, while the blue and red column indicate the elongation (EL) of those specimens. As shown in Figure 8a, only the STOT specimen of T91 steel could not resist creep rupture before the conclusion of the test. Under the test condition of 650 °C/60 MPa, the ICHAZ and FGHAZ specimens of T91 steel were more susceptible to creep rupture (Figure 8b). All of the fractured specimens exhibited high rupture ductility during short-term creep tests. When compared to T91 steel, all of the T92 samples showed high resistance to creep deformation, even under the test condition of 650 °C/60 MPa, which indicate that the creep properties of the simulated T92 steel samples were better than those of the T91 counterpart specimens. 

**Figure 9:**
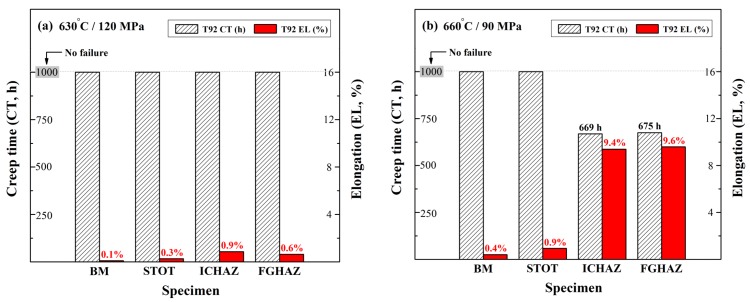
Creep tests of T92 specimens after PWHT at: (**a**) 630 °C/120 MPa; and (**b**) 660 °C/90 MPa.

The results from the short-term creep tests for various T92 specimens under severe creep conditions are shown in Figure 9. Under the test conditions of 630 °C/120 MPa, none of the T92 samples fractured before the test ended. However, the ICHAZ and FGHAZ specimens exhibited slightly higher deformation relative to the BM and STOT specimens (Figure 9a). The greater deformation of the ICHAZ and FGHAZ specimens also reflected their lower creep strengths in comparison to those of the BM and STOT specimens. In the case of the creep condition under 660 °C/90 MPa, the ICHAZ and FGHAZ specimens exhibited large deformation and fractured within 700 h (Figure 9b). In contrast, the STOT specimen was resistant to creep fracture, but deformed a little more than the BM specimen. Clearly, the ICHAZ and FGHAZ specimens with fine-grained structure had a lower creep resistance than the STOT and BM specimens. Inevitably, the HAZs were the inferior regions of a Gr. 92 steel weld and were responsible for the short creep life of the weld.

## 4. Discussion

It has been reported that the A_C1_ and A_C3_ temperatures of Gr. 91 steel increases with an increased heating rate [3]. The dissolution of carbides and lath martensite into austenite is a diffusion-controlled process; thus, a high heating rate will moderately shift the A_C1_ and A_C3_ temperatures to a higher temperature range. The results of this work also showed the same trend of increases in the A_C1_ and A_C3_ temperatures of T92 steel with increasing heating rates. In comparison to the A_C1_ and A_C3_ temperatures of T91 steel at the same heating rate [37], the transformation temperatures of T92 steel were higher than those of T91 steel, especially at the high heating rate of 60 °C/s. Such results could be attributed to the M_23_C_6_ carbides in T92 steel, which are more stable than those in T91 [2,3], causing the delay in carbide dissolution during heating. With regard to the 900 °C/1 min (ICHAZ)-treated samples, the as-heated hardness of T91 steel was Hv 380, which was higher than the Hv 352 of T92 steel. This implies that fewer carbides in T92 steel were dissolved into the matrix during short-time-heating, resulting in lower hardness when compared to T91 steel.

Type IV failure is associated with the enhanced formation of creep voids in the HAZs of 9-12Cr steel welds. For Gr. 91 steel, Type IV cracking may occur in the simulated ICHAZ heated slightly below complete austenization [20], a peak temperature of 925 °C with the finest grain size [22], heated to 875 °C/5 min in a furnace [23]. The creep rupture of the P91 weld is reported to be controlled by the FGHAZ [39], and cracking at the lower-peak temperature of the FGHAZ, or the edge of the HAZ adjacent to the BM [24]. In a prior study [37], the trend of creep rupture of a simulated HAZ sample of Gr. 91 steel was affected by the testing conditions. Fine grained ICHAZ and FGHAZ were more likely to rupture at high temperatures and under low stress, whereas the STOT specimen had low creep resistance relative to other samples at low temperatures and under high stress. The simulated fine grained structure of Gr. 92 steel produced by heating to the A_C3_ temperature shows low creep resistance or minimum time to fracture [4,25,27]. Moreover, the FGHAZ of a P92 steel weld (heated to just above A_C3_) was more likely to creep fracture at higher temperatures and lower stress [28]. In this work on the effect of simulated microstructures of T92 steel on short-term creep life, the results indicated that both the ICHAZ and FGHAZ specimens with fine grained sizes had low resistance to creep rupture, relative to the BM and the STOT specimens.

Over-tempering was expected to cause a recovery of excess dislocations in the tempered martensite, a degraded lath structure, and precipitate-coarsening of Gr. 91 steel [16]. Such alteration of the microstructures contributed to a decrease in the creep strength of 9-12 Cr steels. For 9Cr-1Mo steel [24], the simulated microstructures heated to 865 °C for five minutes were associated with lower strength in the temperature range of ambient to 600 °C in comparison to other samples. Furthermore, the simulated STOT specimen of T91 steel was more likely than other specimens to creep rupture at 615 °C/80 MPa [37]. When compared to the T91 steel, the STOT specimen of T92 steel exhibited a lower tendency for creep rupture than the ICHAZ and FGHAZ specimens in the same creep condition. The addition of B and W into the 9Cr steel improved carbide stability, reduced carbide-coarsening, and retarded dislocation recovery [2,3,4,5,6,7]. With these advantageous characteristics, the short-time over-tempering of T92 steel would have a less detrimental effect on the creep strength of this alloy, as confirmed by the short-term creep tests in this study.

The inherent microstructures of the as-simulated ICHAZ specimen of T92 steel consisted of fresh martensite, ferrite subgrains, and residual M_23_C_6_ carbides. The coarsening of M_23_C_6_ carbides, coalescence of the fine lath structure, and the formation of ferrite subgrains occurred during the PWHT of the ICHAZ specimen of T92 steel. The harmful metallurgical factors of the ICHAZ specimen of T92 steel accounted for its lowest hardness among the samples, as seen in Table 2. The existence of fine ferrites in 9Cr steel should lower its creep resistance; therefore, the fine grain size and degraded microstructures of the ICHAZ specimen were detrimental to its creep resistance. Although the FGHAZ and the BM specimens had the same tempered hardness, the short-time solution treatment at 940 °C led to a fine grain size and undissolved M_23_C_6_ carbides. The coarse M_23_C_6_ carbides in the tempered FGHAZ specimen were expected to be the preferred sites for the nucleation of creep cavities [14]. Thus, the creep-rupture life of FGHAZ specimens of T92 steel was shorter than that of the STOT and BM specimens tested at 660 °C/90 MPa, as shown in Figure 9b. Furthermore, it was noted that the fine grain size was not the cause of the Type IV cracking of 9-12 Cr steel [27,29]; instead, the lack of precipitation-hardening at the boundary and sub-boundary appears to be responsible for the degraded creep strength. Overall, it was expected that the creep lives of the ICHAZ specimen would be shorter than those of the FGHAZ specimen of T92 steel during the long-term creep test due to the degraded microstructures.

## 5. Conclusions

This study experimentally investigated the effects of simulated microstructures similar to those found in the heat-affected zone (HAZ) of a T92 steel weld on their creep rupture at elevated temperatures. The major findings of this study can be summarized as follows:

The A_C1_ and A_C3_ temperatures of the T92 steel determined by a dilatometer at a heating rate of 0.5 °C were respectively 869 °C and 921 °C. Various microstructures in the HAZs were simulated by the infrared heating of the specimens to 860 °C (STOT), 900 °C (ICHAZ), and 940 °C (FGHAZ) for one minute. After PWHT at 750 °C/2 h, the hardness of the BM and FGHAZ specimens were equivalent, whereas the ICHAZ specimen was softer than the other samples. The results of short-term creep tests at 630 °C/120 MPa indicated that the ICHAZ and FGHAZ specimens exhibited slightly higher deformation compared to the BM and STOT specimens, which implied a lower creep strength. In the case of creep condition under 660 °C/90 MPa, the ICHAZ and FGHAZ specimens exhibited great deformation and fractured within 700 h. Furthermore, the creep resistance of the ICHAZ and FGHAZ specimens was lower than that of the STOT and BM specimens. Thus, the simulated T92 steel samples showed higher creep strength than that of the T91 counterpart specimens.

## Figures and Tables

**Table 1:** The transformation temperatures of T92 steel determined by a dilatometer at specific heating/cooling rates.

	Rate (°C/s)	Heating Rate				Cooling Rate			
Temperature (°C)		0.5	15	30	60	5	15	25	45
A_C1_		869	886	903	914				
A_C3_		921	934	945	962				
*M*_s_						392	390	389	374
*M*_f_						229	224	222	195

**Table 2:** The average Vickershardness of the base metal and simulated specimens with or without PWHT at 750 °C/2 h.

Specimen	T92 BM	STOT (860 °C)	ICHAZ (900 °C)	FGHAZ (940 °C)
	VickersHardness (Hv)			
As-treated	245	231	352	400
After PWHT	240	228	223	242

BM = base metal; STOT = short-time over-tempering; ICHAZ = intercritical heat-affected zone; FGHAZ = fine-grained heat-affected zone; PWHT = post-weld heat treatment.

## References

[1] Viswanathan R., Coleman K., Rao U. (2006). Materials for ultra-supercritical coal-fired power plant boilers. Int. J. Press. Vessel. Pip..

[2] Albert S., Kondo M., Tabuchi M., Yin F., Sawada K., Abe F. (2005). Improving the creep properties of 9Cr-3W-3Co-NbV steels and their weld joints by the addition of boron. Metall. Mater. Trans. A.

[3] Das C.R., Albert S.K., Swaminathan J., Raju S., Bhaduri A.K., Murty B.S. (2012). Transition of crack from Type IV to Type II resulting from improved utilization of Boron in the modified 9Cr-1Mo steel weldment. Metall. Mater. Trans. A.

[4] Abe F., Tabuchi M., Kondo M., Okada H. (2006). Suppression of Type IV fracture in welded joints of advanced ferritic power plant steels–effect of boron and nitrogen. Mater. High Temp..

[5] Hong S.G., Lee W.B., Park C.G. (2001). The effects of tungsten addition on the microstructural stability of 9Cr–Mo Steels. J. Nucl. Mater..

[6] Abe F. (2004). Coarsening behavior of lath and its effect on creep rates in tempered martensitic 9Cr-W steels. Mater. Sci. Eng. A.

[7] Otoguro Y., Matsubara M., Itoh I., Nakazawa T. (2000). Creep rupture strength of heat affected zone for 9Cr ferritic heat resisting steels. Nucl. Eng. Des..

[8] Totemeier T.C., Tian H., Simpson J.A. (2006). Effect of normalization temperature on the creep strength of modified 9Cr-1Mo steel. Metall. Mater. Trans. A.

[9] Shrestha T., Alsagabi F.S., Charit I., Potirniche P.G., Glazoff V.M. (2015). Effect of heat treatment on microstructure and hardness of Grade 91 steel. Metals.

[10] Pandey C., Giri A., Mahapatra M.M. (2016). Effect of normalizing temperature on microstructural stability and mechanical properties of creep strength enhanced ferritic P91 steel. Mater. Sci. Eng. A.

[11] Wang S.S., Peng D.L., Chang L., Hui X.D. (2013). Enhanced mechanical properties induced by refined heat treatment for 9Cr-0.5Mo-1.8W martensitic heat resistant steel. Mater. Des..

[12] Robertson D.G., Holdsworth S.R. (2005). European Creep Collaborative Committee (ECCC) Data Sheets 2005.

[13] Robertson D.G., Holdsworth S.R. (2014). European Creep Collaborative Committee (ECCC) Data Sheets 2014.

[14] Brózda J. (2005). New generation creep-resistant steels, their weldability and properties of welded joints: T/P92 steel. Weld. Int..

[15] Caminada S., Cumino G., Lauro A. (2012). Experiences in the use of advanced materials for Ultra Super Critical thermoelectric power plants: ASTM P92 grade and its weldability. Weld. Int..

[16] Zhang Z., Holloway G., Marshall A. (2011). Properties of T/P92 weld metals for ultra super critical (USC) power plant. Int. J. Microstruct. Mater. Prop..

[17] Paul V.T., Saroja S., Hariharan P., Rajadurai A., Vijayalakshmi M. (2007). Identification of microstructural zones and thermal cycles in a weldment of modified 9Cr-1Mo steel. J. Mater. Sci..

[18] Moitra A., Parameswaran P., Sreenivasan P.R., Mannan S.L. (2002). A toughness study of the weld heat-affected zone of a 9Cr-1Mo steel. Mater. Charact..

[19] Mitra A., Siva Prasad N., Janaki Ram G.D. (2016). Influence of temperature and time of post-weld heat treatment on stress relief in an 800-mm-thick steel weldment. J. Mater. Eng. Perform..

[20] Gaffard V., Gourgues-Lorenzon A.F., Besson J. (2005). High temperature creep flow and damage properties of the weakest area of 9Cr1Mo-NbV martensitic steel weldments. ISIJ Int..

[21] Hongo H., Tabuchi M., Watanabe T. (2012). Type IV Creep Damage Behavior in Gr.91 Steel Welded Joints. Metall. Mater. Trans. A.

[22] Milović L., Vuherer T., Blačić I., Vrhovac M., Stanković M. (2013). Microstructures and mechanical properties of creep resistant steel for application at elevated temperatures. Mater. Des..

[23] Divya M., Das C.R., Albert S.K., Goyal S., Ganesh P., Kaul R., Swaminathan J., Murty B.S., Kukreja L.M., Bhaduri A.K. (2014). Influence of welding process on Type IV cracking behavior of P91 steel. Mater. Sci. Eng. A.

[24] Wang Y., Li L. (2016). Microstructure evolution of fine-grained heat affected zone in Type IV failure of P91 welds. Weld. J..

[25] Matsui M., Tabuchi M., Watanabe T., Kubo K., Kinugawa J., Abe F. (2001). Degradation of creep strength in welded joint of 9%Cr steel. ISIJ Int..

[26] Abe F., Tabuchi M. (2004). Microstructure and creep strength of welds in advanced ferritic power plant steels. Sci. Technol. Weld. Join..

[27] Abe F., Tabuchi M., Tsukamoto S., Shirane T. (2010). Microstructure evolution in HAZ and suppression of Type IV fracture in advanced ferritic power plant steels. Int. J. Press. Vessel. Pip..

[28] Xue W., Pan Q., Ren Y., Shang W., Zeng H., Liu H. (2012). Microstructure and type IV cracking behavior of HAZ in P92 steel weldment. Mater. Sci. Eng. A.

[29] Liu Y., Tsukamoto S., Shirane T., Abe F. (2013). Formation Mechanism of Type IV Failure in High Cr Ferritic Heat-Resistant Steel-Welded Joint. Metall. Mater. Trans. A.

[30] Albert S.K., Matsui M., Watanabe T., Hongo H., Kubo K., Tabuchi M. (2002). Microstructural Investigations on Type IV Cracking in a High Cr Steel. ISIJ Int..

[31] Albert S.K., Matsui M., Watanabe T., Hongo H., Kubo K., Tabuchi M. (2003). Variation in the Type IV cracking behaviour of a high Cr steel weld with post weld heat treatment. Int. J. Press. Vessel. Pip..

[32] Haney E.M., Dalle F., Sauzay M., Vincent L., Tournié I., Allais L., Fournier B. (2009). Macroscopic results of long-term creep on a modified 9Cr-1Mo steel (T91). Mater. Sci. Eng. A.

[33] Kimura K., Kushima H., Sawada K. (2009). Long-term creep deformation property of modified 9Cr-1Mo steel. Mater. Sci. Eng. A.

[34] Sawada K., Kushima H., Tabuchi M., Kimura K. (2011). Microstructural degradation of Gr.91 steel during creep under low stress. Mater. Sci. Eng. A.

[35] Zhao L., Jing H., Xiu J., Han Y., Xu L. (2014). Experimental investigation of specimen size effect on creep crack growth behavior in P92 steel welded joint. Mater. Des..

[36] Kim B.J., Ryu S.H., Lim B.S. (2004). Fatigue crack growth behavior of heat affected zone in P92 steel weldment. Met. Mater. Int..

[37] Hsiao T.H., Chen T.C., Jeng S.L., Chung T.J., Tsay L.W. (2016). Effects of simulated microstructure on the creep rupture of the modified 9Cr-1Mo steel. J. Mater. Eng. Perform..

[38] Yu X., Babu S.S., Terasaki H., Komizo Y., Yamamoto Y., Santella M.L. (2013). Correlation of precipitate stability to increased creep resistance of Cr-Mo steel welds. Acta Mater..

[39] El-Azim M.E.A., Ibrahim O.H., El-Desoky O.E. (2013). Long term creep behaviour of welded joints of P91 steel at 650 °C. Mater. Sci. Eng. A.

